# Supplementary material for: Learning curve and surgical outcome of robotic assisted colorectal surgery with ERAS program
Source: Sci Rep. 2022 Nov 29;12:20566. doi: 10.1038/s41598-022-24665-w (PMC9709162; doi:10.1038/s41598-022-24665-w)
Supplement: Supplementary file 1 — Supplementary Information 1. [file 41598_2022_24665_MOESM1_ESM.zip › LSC Raw data-Ñ[▒K/140/P_ñw╝╨░O▒Kñσ.pdf]

[redacted]

Pathologic diagnosis:
Colon, terminal ileum, and appendix, right hemicolectomy --- a. S/P EMR without residual malignancy. b. Tubular adenoma. c. Unremarkable lymph node (0/17).

Gross description:
The specimen consists of a segment of colon, 19 cm in length, and 3.5 cm in proximal circumference, 2 cm in distal cut end circumference, and a segment of terminal ileum, 5.5 cm in length, and 2 cm in cut end circumference. A hematoma, 3.5x3x2 cm, is seen near the ileocecal valve. A clip is seen, cm to the distal cut end. On cross section, no defenite invasion is seen. A polyp, 0.2x0.2x0.2cm, is seen 4.8 cm to the ileocecal valve. The appendix, ileocecal valve, and both cut ends are free of tumor. Representative part for section: A,B) distal cut end and mucosa around the clip C) radial margin D) proximal cut end E) ileocecal valve F) polyp G-J) hematoma and appendix K-M) lymph nodes.

#T-59420_2 #M-43000_2 0 1105 000000
#T-C4560_2 #M-09183_2 0 1105 000000
#T-59200_2 #M-09183_2 0 1105 000000

已電子簽章

敬告：本單僅供醫師參考使用，不具任何申請效力！！
請病患妥為保管，若不慎遺失需自負個人資料外洩責任！
